# Relationship between the Chemical Composition and the Biological Functions of Coffee

**DOI:** 10.3390/molecules26247634

**Published:** 2021-12-16

**Authors:** Shah Saud, Ahmad Mohammad Salamatullah

**Affiliations:** 1 College of Life Sciences, Linyi University, Linyi 276012, China; saudhort@gmail.com; 2 Department of Food Science & Nutrition, College of Food and Agricultural Sciences, King Saud University, Riyadh 11451, Saudi Arabia

**Keywords:** coffee, chemical constituents, biological activities, phenolic acids, flavonoids

## Abstract

Coffee is a Rubiaceae coffee plant ranked as the first of the three most important beverages in the world, with effects including lowering blood sugar, protecting the liver, and protecting the nerves. Coffee contains many chemical components, including alkaloids, phenolic acids, flavonoids, terpenoids, and so on. Chemical components in coffee are the basis of its biological function and taste. The chemical components are the basis of biological activities and form the characteristic aroma of coffee. The main chemical components and biological activities of coffee have been extensively studied, which would provide a relevant basis and theoretical support for the further development of the coffee industry.

## 1. Introduction 

Coffee is a Ribinaceae coffea plant that is native to north-central Africa and is mainly found in some countries in South and Central America, Africa, and Asia. There are 4 groups of 66 species in the genus *Coffea*. The coffees are generally large-grain species (*C. liberica*), medium-grain species (*C. robusta*), small-grain species (*C. arabica*), and excelsa species (*C. excelsa*) of Eucoffea. [1]. In 1892 French missionaries introduced coffee growing in China’s Yunnan Province and continued to expand production. Currently China’s main coffee is grown in Yunnan and on Hainan Island, and more than 99% of coffee grown in Yunnan is small seed coffee, categorized by the international coffee organization expert rating of quality coffee in the world [2]. Coffee represents a class of complex structures and mixtures of brown and macromolecular polymers formed by the Maillard reaction of carbonyl and amino compounds in coffee beans during the roasting of the beans [2,3], which have antioxidant properties [4], antibacterial properties [5], anti-hyperlipidemia [6], and anti-caries. Coffee can also improve intestinal microenvironment and other biological activities [7]. In addition, “polyphenols”, which are potent antioxidant compounds, can be brought into oxidative stress and chronic inflammation. Many studies have focused on their beneficial anti-inflammatory, analgesic and antibacterial, vasodilator, anti-allergic, and anti-cancer effects. Recent studies have shown that the beneficial effects are also related to the ability of polyphenols to interact with major cell signaling and gene regulation pathways and to regulate the intestinal microbiota. For example, polyphenols can affect the F/B ratio by inhibiting the growth of specific bacterial species. In fact, a series of pharmacological effects of different phenolic compounds (especially flavonoids) have been proved through in vitro, in vitro and animal experiments. However, the health effects of these compounds depend on their bioavailability, and it is also important that they are absorbed, metabolized, and eliminated from the body.

In addition, according to Chinese material medica, coffee is slightly bitter, astringent and flat. It has the effect of causing the drinker to wake up, and can also cause diuresis and strengthening of the stomach. It is used for awakening, diuresis, and strengthening the stomach. According to research reports, the chemical components contained in coffee mainly include alkaloids, phenolic acids, flavonoids, terpenoids, sterols, and volatile components, which have a variety of pharmacological effects such as insulin sensitization, improvement of sugar metabolism, anti-diabetes, and liver protection effects. In order to provide theoretical support for further development of coffee industry, the sources and biological activities of chemical components in coffee were reviewed in this paper.

## 2. Main Ingredients of Coffee

### 2.1. Chemical Component

The chemical composition of coffee beans is quite complex, and carbohydrates account for the most components. Coffee beans contain a variety of carbohydrates, accounting for 60% of the total weight of raw coffee beans. There are also some proteins, fats, tannins, caffeine, minerals, and other trace ingredients. Variety, origin, and harvest season will affect the composition of these ingredients. The various ingredients of raw coffee beans react chemically during roasting, forming the unique flavors and colors of various coffee beans [8]. Table 1 shows the main chemical composition of raw coffee beans.

### 2.2. Volatile Components of Coffee

Volatile components are the core that affects the taste of coffee. Raw beans do not contain the special aroma of coffee. After roasting, a variety of flavorings are created. More than 800 kinds of coffee aroma components have been analyzed and there are many kinds of coffee volatile substances. The source is the small molecular derivatives created by the cracking of the bond and the reaction of the chemical components in the beans during roasting, and there are also reactions between the components. The typical ones are formed by the action of basic acids, organic acids, phenols, and sugars to create the flavor of coffee. Oxygen-containing, nitrogen-containing or sulfur-containing ring compounds such as furan, thiophene, pyrazine, thiazole, pyrrole, and pyridine still have some terpene carbonyl and phenol compounds [9]. Recently, solid phase micro-extraction gas chromatography-mass spectrometry (SPME GC/MS) was used to analyze the constituents of green coffee beans and a total of 131 compounds were identified, of which 91 types of compounds were detected with Waning coffee and 106 types of connections were discovered by Chengmai Coffee (with a total of 66 types of connections). The content of total ingredients is 75.52% of the total content [10]. The steam distillation part of coffee oil uses GC / MS to identify 57 components, of which the volatile flavor components can be divided into 12 categories: 3 aldehydes, 11 furans, 14 phenols, 3 thiazoles, 9 olefins, 2 alkanes, 2 esters, 3 ketones, 1 pyrrole, and 4 thiophenes. There are three types of dicarboxylic acids and three types of pyrazines, mainly furans, phenols, and olefins [11]. The volatile components of Laotian coffee were extracted with ultrasound-assisted extraction of n-hexane, dichloromethane and methanol and 77 components were analyzed by GC / MS, including 2 alcohols, 10 phenols, 2 ethers, 3 aldehydes, 12 ketones, 12 acids, 6 acids, 11 esters, 6 hydrocarbons, and 25 nitrogenous oxides [12]. In recent years, various modern techniques have been used to analyze the volatiles of coffee, involving different parts of the coffee, and they have never been interrupted.

### 2.3. Melanoidins Content of Coffee

The content of melanoidins that were extracted from different degrees of roasting Yunnan Arabica rose with increasing degrees of roasting (37, 45 and 46 volatile components from light–roasting–medium–roasting and dark–roasting–degrees). Yunnan Arabica coffee melanoidins were each identified 2-furanmethanol, furfural, 5-hydroxymethylfurfural caffeine hexahydro-3-(Isobutyl) 1,2-a-pyrazine-1,4-dione made up the majority of these components, while the content was different under different baking conditions [13].

### 2.4. Alkaloids

Caffeine (1,3,7-trimethylxanthine, “Caffeine”) is the main alkaloid component in coffee fruits and the source for bitter taste of coffee. Caffeine is widely found in tea, cocoa and coffee, and is one of the widely used psychotropic drugs. Studies have shown that caffeine can relieve the amnesia induced by memory loss in elderly. It can also reduce the risk of neurodegenerative diseases such as Alzheimer’s disease (AD) [14] and Parkinson’s disease (PD) [15,16] showed that moderate caffeine intake could inhibit memory impairment in rats, and [6] confirmed that caffeine may protect AD by promoting the survival of brain striatum and cortex cells and inhibiting the apoptosis pathway. [17] proved by a PD model of human bone marrow neuroblastoma cell line (SH-SY5Y) that caffeine can reduce caspase-3 activity of cysteine and reduce the number of nuclear fragments and apoptotic condensation. Caffeine can also reduce the leakage of blood brain barrier (BBB) caused by 1-methyl-4-phenyl-1,2,3,6-tetrahydropyridine (MPTP) and inhibit the dysfunction of BBB [18]. Regular doses of caffeine can help ameliorate mild hemiplegic stroke. [19] reported that caffeine has a protective effect on stroke through its antioxidant and anti-inflammatory properties caffeine has also been associated with diabetes control [20].

Chemical CoTrigonelline is a pyridine derivative found in several types of fruits and seeds, including coffee. It is found in both Arabica and Robusta coffees in an average amount of 1%. It is severely degraded during coffee roasting and when dark roasting conditions are used only about 0.1 to 0.2% remains in the roasted coffee component. For example, N-methylpyridine (NMP) is a thermal degradation product of trigonelline during coffee roasting [21]. In recent years, many researches on trigonelline, and its biological value is more and more prominent. [22] reported that trigonelline can regulate inflammation by lowering blood glucose and increasing the expression of insulin *β* cells, by down-regulating the expression of apoptotic protease 3, the partial apoptosis of *β* cells was inhibited and the activity of antioxidant enzyme was increased, thus the protective effect on type 1 diabetic mice was obtained. Ginsenoside Rb1 and trigonelline can regulate mir-3550 and act on Wnt/*β-*catenin signaling pathway to prevent the development of diabetic kidney injury [23]. Trigonelline has a potential therapeutic effect on the heart tissue of colitis [24]. Trigonelline has a neuroprotective effect and is a good drug for the treatment of neurodegenerative diseases. [25,26] reported that trigonelline can improve cognition and relieve neural loss. It can prevent liver lipid accumulation and lipotoxicity caused by high cholesterol and high fat diet by restoring liver autophagy [27]. It also inhibits choline intestinal microbial metabolism and its associated cardiovascular risks [28].

In addition, coffee contains theobromine, theophylline, and nicotinic acid [29] reported that there are also 1,3,7,9-tetramethyluric acid (“theacrine”), liberine and methyllibetine in coffee leaves (Table 2).

### 2.5. Phenolic Acids and Their Derivatives

At present, p-hydroxybenzoic acid, vanillic acid, *P*-coumaric acid, ferulic acid and chlorogenic have been separated from coffee Acid, Caffeic acid, Caffeoylquinic acid, Dicaffeoylquinic acid, 3-*O*-feruloylquinic acid, Caffeic acid, Caffeoylquinic acid, 3-*O*-Feruloylquinic acid Acid), 3-*O*-ferulic acid-4-*O*-coffee acyl quinic acid (3-Oferuloyl-4-*O*-caffeoylquinic acid), 3-*O*-coffee acyl-4-*O*-ferulic acid acyl quinine (3-*O*-caffeoyl-4-*O*-feruloylquinic Phenolic acids and caffeic acid derivatives (Table 3).

Chlorogenic acid (CGA) is the main phenolic acid compound, which has the biological functions of lowering blood lipid, antioxidant and antibacterial [32] reported that chlorogenic acid can significantly reduce cholesterol, triglyceride, low density lipoprotein and increase high density lipoprotein [33] confirmed the activity and DNA protection of chlorogenic acid [34] reported that chlorogenic acid can reverse transcription cort-induced autophagy and apoptosis of PC12 cells and also regulate the AKT/mTOR pathway of PC12 cells [35] reported that chlorogenic acid can inhibit the intracellular metabolism of pseudomonas aeruginosa P1 cells and play an antibacterial role. Chlorogenic acid can effectively reduce the absorption and accumulation of Cd in jejunum and protect the intestinal barrier [36]. Blood glucose test for 2 h of oral glucose in human confirmed that chlorogenic acid and trigonelline can reduce the early glucose and insulin response [37]. At the same time, the thermal degradation of CGA during coffee roasting leads to the formation of bitter phenolic compounds and phenolic aromatic compounds. CGA also participates in the formation of coffee color through the framework of protein melanin incorporation, which is the main cause of coffee pigmentation and astringency.

**Table 3:** Phenolic acid compounds in coffee.

No.	Compound	Species	Part	Reference
9	Vanillic Acid	CA, CR	L	[38]
10	Benzoic Acid	CA, CR	L	[38]
11	*p*-Hydroxybenzoic Acid	CA, CR	L	[38]
12	3-Hydroxybenzoic Acid	CA, CR	L	[38]
13	Gentosic Acid	CA, CR	L	[38]
14	Protocatechuic Acid	CA, CR	L	[38]
15	Caffeic Acid	CA, CR	GB, CB	[39]
16	Sinapic Acid	CA, CR	L	[38]
17	Ferulic Acid	CR	GB, L	[38]
18	*p*-Coumaric Acid	CA	GB, L	[38]
19	Caftaric Acid	CA, CR	L	[38]
20	3-*O*-*p*-Coumaroylquinic Acid	CA, CR	GB, CB	[39]
21	5-*O*-*p*-Coumaroylquinic Acid	CA, CR	GB, CB	[39]
22	4-*O*-*p*-Coumaroylquinic Acid	CA, CR	GB, CB	[39]
23	3-*O*-Caffeoylquinic Acid CA, CR		GB, CB	[39]
24	4-*O*-Caffeoylquinic Acid	CA, CR	GB, CB	[39]
25	5-*O*-Caffeoylquinic Acid	CA, CR	GB, CB	[39]
26	1-*O*-Caffeoylquinic Acid	CA, CR	CB	[39]
27	1-*O*-Caffeoylquinic Acid Methyl Ester	CR	GB	[39]
28	3-*O*-Caffeoylquinic Acid Methyl Ester	CA, CR	GB	[39]
29	5-*O*-Caffeoylquinic Acid Methyl Ester	CA, CR	GB	[39]
30	3,4-di-*O*-Caffeoylquinic Acid	CA, CR	GB, CB	[39]
31	3,5-di-*O*-Caffeoylquinic Acid	CA, CR	GB, CB	[39]
32	4,5-di-*O*-Caffeoylquinic Acid	CA, CR	CB	[39]
33	3,4-di-*O*-Caffeoylquinic Acid Methyl Ester	CA, CR	GB	[39]
34	3,5-di-*O*-Caffeoylquinic Acid Methyl Ester	CA, CR	GB	[39]
35	4 4,5-di-*O*-Caffeoylquinic Acid Methyl Ester	CA, CR	GB	[39]
36	3-*O-*Feruloylquinic Acid	CA, CR	GB, CB	[39]
37	4-*O-*Feruloylquinic Acid	CA, CR	GB, CB	[39]
38	5-*O-*Feruloylquinic Acid	CA, CR	GB, CB	[39]
39	1-*O*-Feruloylquinic Acid Methyl Ester	CA, CR	GB, CB	[39]
40	3-*O*-Feruloylquinic Acid Methyl Ester	CA, CR	GB, CB	[39]
41	5-*O*-Feruloylquinic Acid Methyl Ester	CA, CR	GB, CB	[39]
42	3,4-di-*O*-Feruloylquinic Acid	CR	GB	[39]
43	3,5-di-*O*-Feruloylquinic Acid	CR	GB	[39]
44	4,5-di-*O*-Feruloylquinic Acid	CR	GB	[39]
45	3-*O*-Feruloyl-5-*O*-Caffeoylquinic Acid	CA, CR	GB, CB	[39]
46	3-*O*-Feruloyl-4-*O*-Caffeoylquinic Acid	CA, CR	GB, CB	[39]
47	4-*O*-Feruloyl-5-*O*-Caffeoylquinic Acid	CA, CR	GB, CB	[39]
48	3-*O*-Caffeoyl-4-*O*-Feruloylquinic Acid	CA, CR	GB, CB	[39]
49	3-*O*-Caffeoyl-5-*O*-Feruloylquinic Acid	CA, CR	GB, CB	[39]
50	4-*O*-Caffeoyl-5-*O*-Feruloylquinic Acid	CA, CR	GB, CB	[39]
51	3-*O*-Feruloyl-4-*O-p*-Coumaroylquinic Acid	CA, CR	GB	[39]
52	3-*O*-*p*-Coumaroyl -*5*-*O*-Feruloyl Quinic Acid	CA, CR	GB	[39]
53	3-*O*-Caffeoyl-5-*O*-*p*-Coumaroylquinic Acid	CR	GB	[39]
54	4-*O*-*p*-Coumaroyl-5-*O*-Caffeoylquinic Acid	CA, CR	GB	[39]
55	4-*O*-Caffeoyl-5-*O*-*p*-Cumaroylquinic Acid	CR	GB	[39]
56	Caffeoyl-*N*-Tryptophan	CA, CR	GB, CB	[39]
57	*p*-Coumaroyl-*N*-Tryptophan	CR	GB, CB	[39]
58	Feruloyl-*N*-Tryptophan	CR	GB	[39]
59	5-*O*-Caffeoyl-1,3-Quinide	CA, CR	CB	[39]
60	3-*O*-Caffeoyl-1,5-Quinide	CA, CR	CB	[39]
61	4-*O*-Caffeoyl-1,3-Quinide	CA, CR	CB	[39]
62	5-*O*-Caffeoyl-1,4-Quinide	CA, CR	CB	[39]
63	4-*O*-Caffeoyl-1,5-Quinide	CA, CR	CB	[39]
64	5-*O*-Feruloyl-1,3-Quinide	CR	CB	[39]
65	3-*O*-Feruloyl-1,5-Quinide	CA, CR	CB	[39]
66	4-*O*-Feruloyl-1,3-Quinide	CR	CB	[39]
67	4-*O*-Feruloyl-1,5-Quinide	CR	CB	[39]
68	3,4-di-*O*-Caffeoyl-1,5-Quinide	CA, CR	CB	[39]
69	4,5-di-*O*-Caffeoyl-1,3-Quinide	CA, CR	CB	[39]
70	3-*O*-Caffeoyl-4-*O*-3-Methylbutanoylquinic Acid	CA	GB	[40]
71	3-*O*-Caffeoyl-4-*O*-3-Methylbutanoyl-1,5-Quinide	CA	GB	[40]

### 2.6. Flavonoids

Flavonoids are a kind of active ingredients widely existing in natural plants, with antioxidant, anticancer, anti-inflammatory and antibacterial activities [38,39,40,41,42]. Small seed coffee contains flavonoids such as catechin, epicatechin, and quercetin (Table 4).

### 2.7. Terpenoids

Coffee contains a large number of terpenes, mainly p-kauri type and coffeol diterpenes, among which the contents of coffeol and 16-*O*-methyl coffeol are the highest. 16-*O*-methylcafeol has been used as a marker to distinguish small and medium coffee [45] detected 16-*O*-methylcafestol and 16-*O*-methylkahweol in small coffee roasting beans for the first time by using 600 MHz NMR and LC-MS. In recent years, [45,46,47] isolated and identified four new pairs of mascarosides I~II (mascarosides I~II, paniculoside VI and ofaryloside I), 1 pair of villanovane I and 7 pairs of kauri diterpenoid glycosides; five mascaroside III-V and 20-nor-Cofarylosiii diterpenoids; 8 which showed no inhibition on HL60, A549, SMMC-7721, McF-7 and SW480 tumor cell lines and were isolated from green beans. Among them, caffarolides C, D, and F showed platelet aggregation activation activity in vitro. 3 × 10^−4^ g/mL, respectively (11.4 ± 5.5)%, (15.8 ± 5.6)% (7.8 ± 3.3)%. Four pairs of conuri diterpenes (caffruenol A, caffruenol B, caffruolide A and caffruolide B) were isolated from sun-dried beans and inhibited production in 264.7 macrophages induced by lipopolysaccharide. Triterpenes are also found in coffee. [46] isolated four new dammarane triterpenes (caffruones A~D) from dried fruits of Yunnan coffee for the first time. These results greatly enriched the types of terpenoids in coffee, and provided a lot of reference for the further study of Yunnan coffee.

At present, the studies on the terpenoids of coffee mainly focus on caffein and coffee bean alcohol [47] have reported that cafeol acetate and cafeol had a dose-dependent inhibitory effect on human prostate cancer cells. [48] reported that cafitol inhibits breast cancer cell proliferation and induces cell death by inducing a caspase 3-dependent pathway [49] studied the effects of caffein on NB4, K562, HL60 and KG1 leukemia cell lines, and the results showed that caffein had the highest cytotoxicity to HL60 and KG1 cells, and could reduce the proliferation of HL60 cells by 100% [50] showed that caffein concentrations of 10^−8^ mol/L and 10^−6^ mol/L can increase insulin secretion by 12% and 16%, respectively, and long-term exposure can increase insulin secretion by 34% and 68%. Glucose uptake by human skeletal muscle cells can be significantly increased by 8% [51] reported that cafitol can reduce the production of lipopolysaccharide induced interleukin 1α, 1*β*, 6 and tumor necrosis factorα, and inhibit lipopolysaccharide induced liver inflammation flavonoid compounds in coffee (Table 5).

### 2.8. Flavor Substances

Studies on flavor compounds in coffee beans began in the 1960s, and the correlation between flavor precursors in raw coffee beans and aroma components in roasted coffee was reported in the 1970s [56]. Cell wall polysaccharides, lipids, proteins, sucrose, chlorogenic acid, caffeine, and trigonelline are the main storage compounds of mature coffee seeds. These compounds mainly form coffee flavor through the Maillard reaction, Strecker degradation, and the caramelization reaction during roasting. There are 28 characteristic flavor substances of coffee [57]: (1) aldehydes and ketones are related to caramel/sweet taste: Iso-butyral, 2-methyl-butyral, iso-valeraldehyde, 2,3-butanedione, 2,3-pentanedione, 4-hydroxy-2, 5-dimethyl-3 (2H)-furanone,5-ethyl-4-hydroxy-2-methyl-3 (2H)-furanone and vanillin; (2) Sulfur compounds are associated with sulfur/roasting odor: 2-furfuryl mercaptan, 2-methyl-3-furfuryl mercaptan, 3-methyl-thiopropyl aldehyde, 3-thio-3-methyl-butyl formate, 3-methyl-2-butene-1-mercaptan, methyl-mercaptan and dimethyl trisulfide compounds; (3) pyrazine compounds were related to soil odor: 2-ethyl-3,5-dimethylpyrazine, 2-ethyl-3,5-dimethylpyrazine, 2,3-diethyl-5-methylpyrazine, 2-ethyl-3-ethyl-5-methylpyrazine and 2-methoxy-3-isobutyl pyrazine; (4) Phenols and aldehydes are associated with smoky/phenolic aromas: guaiacol, 4-ethyl guaiacol, 4-vinylguaiacol, acetaldehyde, propanal and (F)-*β* -damarone; and (5) the furan ketone class of compounds associated with pungent taste: 3-hydroxy-4,5-dimethyl-2 (5H)-furanone and 3-hydroxy-4-methyl-5-ethyl-2 (5H)-furanone [58] identified it from steam distillation of coffee oil aldehydes, furans, phenols, thiazoles, alkenes, alkanes, ester, ketone, pyrrole, thiophene, carboxylic acid, pyrazine main 12 volatile components. [59] from Lao coffee including alcohols, phenols, ethers, aldehydes, ketones, acids, 77 volatile components of esters, hydrocarbons and nitrogen oxides. [60] used gas chromatography-mass spectrometry (GC-MS) technology to medium grain 101 volatile compounds have been identified from coffee flowers, from coffee beans a total of 72 volatile compounds were identified.

### 2.9. Other Ingredients

In addition, coffee also contains anthranone compounds mangiferin and isomangiferin, coumarin compounds scopoletin, carotenoids, and lutein compounds [31]. Scorodocarpines D~F is also found in ripe coffee beans [61]. Coffee seeds contain β-sitosterol, stigmasterol, campesterol, cholesterol, Δ5-avenasterolnd, 7Δ-avenasterol, and Δ7-stigmastenol, etc. [62]. Lipids mainly include nutmeg acid, palmitic acid, stearic acid, oleic acid, linoleic acid, arachidonic oil, etc. [62].

## 3. Bioactivity of Coffee

Coffee contains a large number of bioactive substances, with antioxidant, lipid-lowering, hypoglycemic, neuroprotective, and other biological activities.

### 3.1. Antioxidant Activity

Plant phenols are a large and diverse group, including cinnamic acid, benzoic acid, flavonoids, anthocyanins, stilbenes, coumarins, lignan compounds and lignins with different properties [60,61,62,63]. In in vitro tests, plant phenols are known to have strong antioxidant activity. Based on this, it is speculated that plant phenols may protect cell DNA and prevent free radical damage in the body. Since free radicals play an important role in inducing cardiovascular and cancer diseases, the consumption of plant polyphenols can effectively prevent the occurrence of such diseases.

A recent review report claims that four out of five epidemiological observational studies show that flavonols can prevent heart disease, but only one out of five studies show that it has the effect of preventing cancer. Therefore, the evidence obtained for the prevention of cancer by flavonols is obviously unconvincing. In addition, other types of plant polyphenols also need to be further investigated.

### 3.2. Lipid-Lowering Effect

3T3-L1 adipocytes were used to evaluate the effects of coffee fruits of different colors (green, yellow and red) on adipogenesis and/or lipolysis, and the results showed that green coffee fruits of different colors all had the activity of inhibiting adipogenesis in 3T3-L1 adipocytes [64]. Dried red coffee reduced fat accumulation by about 47%. In addition, all the main components of coffee extract (malic acid, quinic acid, and chlorogenic acid) except yellow fresh coffee increased the release of glycerol. At the same time, studies have also confirmed that coffee pulp can reduce cholesterol in vitro and in vivo by down-regulating LXRα activity modulated by NPC1L and inhibiting intestinal cholesterol absorption by micellar complex formation [65].

### 3.3. Lowering Blood Sugar

Diabetes mellitus (DM) is a chronic disease in which blood sugar levels increase due to relative or absolute lack of insulin. Drug therapy and diet management are the main treatments for diabetes. Caffeine, chlorogenic acid, trigonelline and other main components in coffee all have the effect of lowering blood sugar. The authors in [66] conducted a hypoglycemic effect study on small coffee and its leaf ethanol extract, and found that coffee extract can significantly reduce the blood sugar content of mice. Caffeol has potential anti-diabetic effects which can increase glucose-stimulated insulin secretion and increase the uptake of glucose by human skeletal muscle cells [67].

### 3.4. Neuroprotection

**Figure 1:**
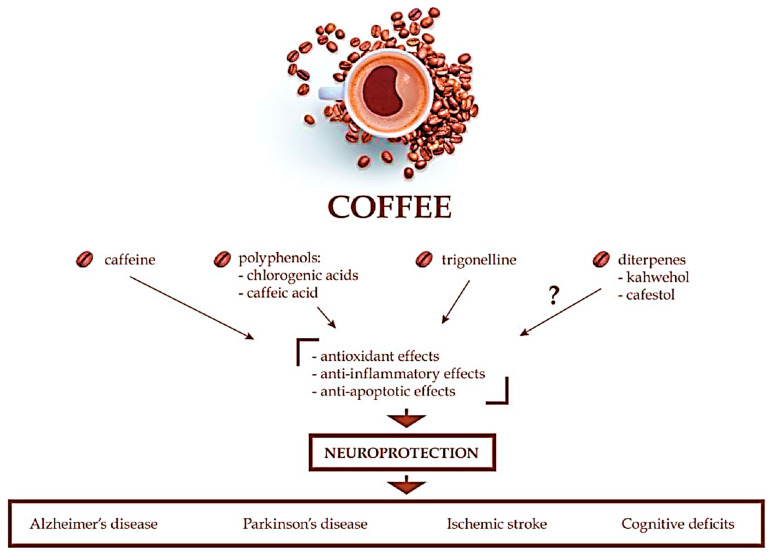
Summary of the neuroprotective effects of coffee [1]. (Socała 2021).

Epidemiological studies have shown that habitual coffee consumption may reduce the risk of Alzheimer’s disease [68], and the coffee intake of male patients with primary Parkinson’s disease is negatively correlated with the severity of tremor [69]. In the APP/PS2 transgenic mouse model of Alzheimer’s disease in Figure 1, However, many reports have shown that many compounds in coffee can independently have neuroprotective effects, suggesting that decaffeinated coffee is also effective against neurodegenerative diseases. Polyphenolic acids (i.e., chlorogenic and caffeic acids) and trigonelline appear to be the most promising, but unlike caffeine, there is a lack of epidemiological studies or clinical reports on their protective effects in neurodegenerative diseases.

### 3.5. Inflammatory, Cardiovascular Activity and Effects of Coffee on Sleep Wakefulness Cycle

The crude extract of coffee peel can protect and restore damaged human umbilical vein endothelial cells to a certain extent [70]. The combination of coffee extract and vitamin C can play an anti-tumor role [71]. The methanol extract of green coffee bean has certain anti-inflammatory activity [72]. Coffee has a protective effect on the liver, and coffee consumption can reduce the risk of HCC recurrence and increase the chance of survival after orthotopic liver transplantation [73]. Coffee was negatively associated with the risk of nonalcoholic fatty liver disease [74]. Studies suggest that the caffeine in a cup of coffee in the morning can not only keep you awake [75], but also help suppress inflammation, which is linked to risk factors for heart disease. Researchers have discovered an inflammatory response mechanism in some elderly people. It is produced in the human body, but not in elderly people. When it is heavily activated, people often have high blood pressure and severe atherosclerosis [76]. In laboratory tests, however, it has been shown that caffeine blocks this inflammatory process and that coffee also has a certain effect on the cardiovascular system. It is mainly caused by stimulating the sympathetic nerves of the heart and causing arousal. Symptoms such as tightness in the chest. The normal heart is innervated by the sympathetic and parasympathetic nervous systems [77]. When it is dormant at night, the parasympathetic nerve is usually the main stimulus. At this point it manifests as bradycardia and relatively low blood pressure. Try sympathetic nerve control during the day. Especially during physical exertion, emotional excitement and when drinking coffee, drinking, smoking and tea, the sympathetic nerves are stimulated to excite them, which leads to palpitations and tightness in the chest.

## 4. Summary and Future Perspectives

As the first of the world’s three major drinks, coffee is closely linked to our daily life. The study of the chemical composition of coffee is an important link to further develop and enhance the utilization of coffee. The lack of brand effect and low added value are the main problems facing China’s coffee industry. In order to solve this dilemma faced by China’s coffee industry, China’s coffee processing enterprises actively explore the deep processing technology of coffee, and strive to transform the resource advantage into economic advantage. However, the change of this situation must be based on the in-depth study of coffee.

(1) The rich chemical composition of coffee is the key to affecting the biological activity and flavor of coffee. Therefore, the study of its chemical composition will be the basis to further improve and promote the research of coffee, and also the key to improve the flavor of coffee.

(2) Through a comprehensive and in-depth study of coffee, comprehensive utilization will be an important link for further development and utilization of coffee, such as coffee flowers, coffee leaves, coffee grounds, etc. The coffee flower contains phenols, caffeine and trigonelline, and other active substances with antioxidant capacity, and has the potential to transform into biological sugars. Secondly, coffee leaves contain less caffeine and can be used as a tea substitute.

(3) Yunnan coffee is of good quality and its diterpenoids are unique. Therefore, it is very important to explore the relationship between its chemical composition and flavor to improve the quality of coffee.

## Figures and Tables

**Table 1:** The main chemical composition of raw coffee beans.

Ingredient Content (%)	
Carbohydrates 60.0	(1)
Reducing sugar 1.0	(2)
Sucrose 7.0	(3)
Pectin 2.0	(4)
Starch 10.0	(5)
Pentan 5.0	(6)
Hemicellulose 15.0	(7)
Whole cellulose 18.0	(8)
Lignin 2.0	(9)
Grease 13.0	(10)
Protein (N*6.25) 13.0	(11)
Ash content (oxide) 4.0	(12)
Tannic acid 13.0	(13)
N-methylnicotinic acid (soluble) 1.0	(14)
Caffeine (soluble) 1.0~~2.0	(15)

**Table 2:** Alkaloid compounds in coffee.

No.	Compound	Species	Part	Reference
1	Caffeine	CA, CR	L, GB, CB Bean	[30,31]
2	Theobromine	CA, CR	CB	[30]
3	Theophyline	CA, CR	CB	[30]
4	1,3,7,9-Theacrine	CL	L	[29]
5	Liberine	CL	L	[29]
6	Methyllibetine	CL	L	[29]
7	Trigonelline	CA, CR	GB, CB	[30,31]
8	Nicotinic acid	CA, CR	CB	[30]

CA, *Coffea arabica*; CR, *C. robusta*; CL, *C. liberica*; L, leaf; GB, green bean; CB, roasted bean. The same is true of the abbreviations in the following tables.

**Table 4:** Flavonoids compounds in coffee.

No.	Compound	Species	Part	Reference
72	Catechin	CA	L	[43]
73	Epicatechin	CA	L	[43]
74	Epicatechin gallate	CA	L	[43]
75	Epigallocatechin gallate	CA	L	[43]
76	Delphinidin-3,5-dilucoside	CA	L	[43]
77	Delphinidin-3-(6ʹʹ-malonyl-glucoside)	CA	L	[43]
78	Cyanidin-3-*O*-glucoside	CA	P	[43]
79	Cyanidin-3-*O*-Rutinoside	CA	P	[43]
80	Kaempferol	CA	L	[44]
81	Kaempferol-3-Glc	CA	L	[44]
82	Kaempferol-3-Glc-Hex-DeHex	CA	L	[44]
83	Kaempferol-3-Glc-Hex	CA	L	[44]
84	Kaempferol-3-Glc-(6ʹʹ-Rha)	CA	L	[44]
85	Quercetin	CA	L	[44]
86	Quercitrin	CA	L	[44]
87	Isoquercitrin	CA	L	[43]
88	Rrutin	CA	L	[44]
89	Hyperoside	CA	L	[44]
90	Quercetin-3-Glc-Hex-DeHex	CA	L	[44]
91	Quercetin-3-glucuronide	CA	L	[44]
92	Luteolin	CA	L	[44]
93	Patuletin	CA	L	[44]
94	Fisetin	CA	L	[44]
95	Myricetin	CA	L	[44]
96	pigenin	CA	L	[44]

SB, sun bean.

**Table 5:** Terpenes compounds in coffee.

No.	Compound	Species	Part	Reference
97	Ursolic Acid	CA	L	[31]
98	Caffruone A	CA	SB	[46]
99	Caffruone B	CA	SB	[46]
100	Caffruone C	CA	SB	[46]
101	Caffruone D	CA	SB	[46]
102	Caffruenol A	CA	SB	[42]
103	Caffruenol B	CA	SB	[42]
104	Caffruolide A	CA	SB	[42]
105	Caffruolide B	CA	SB	[42]
106	Tricalysiolide A	CA	SB	[42]
107	Tricalysiolide B	CA	SB	[42]
108	Tricalysiolide C	CA	SB	[42]
109	Tricalysiolide E	CA	SB	[36]
110	16*α*,17-Dihydroxy-ent-kauran-19-al	CA	SB	[42]
111	16*β*,17-Hydroxy-ent-kauran-19-oic Acid	CA	SB	[42]
112	16*α*,17-Dihydroxy-ent-kauran-19-oic Acid	CA	SB	[42]
113	9*β*,16*α*,17-Trihydroxy-ent-kauran-19-oic Acid	CA	SB	[42]
114	16*β*-7,17-Dihydroxy-ent-kauran-19-oic-Methyl Ester	CA	SB	[42]
115	16α,17-Dihydroxy-9(11)-ent-kauren- 19-oic Acid	CA	SB	[42]
116	(2*β*,4*β*,15*α*)-15-Hydroxy-2-{[2-*O*-(3-methyl-1-oxo-butyl)]-*β*-D-glucopyrnosyl]oxy}-18-nor-ent kaur-16 en-18-oic Acid	CA	SB	[42]
117	Caffarolide A	CA	GB	[43]
118	Caffarolide B	CA	GB	[43]
119	Caffarolide C	CA	GB	[43]
120	Caffarolide D	CA	GB	[43]
121	Caffarolide E	CA	GB	[43]
122	Caffarolide F	CA	GB	[43]
123	Caffarolide G	CA	GB	[43]
124	Caffarolide H	CA	GB	[43]
125	Mascaroside I	CA	GB	[44]
126	Mascaroside II	CA	GB	[44]
127	Paniculoside VI	CA	GB	[44]
128	Cofaryloside I	CA	GB	[44]
129	Villanovane I	CA	GB	[44]
130	Mozambioside	CA	GB	[44]
131	Bengalensol	CA	GB	[44,45]
132	19-Norkaur-16-en-18-oic acid-15-hydroxy-2-[[2-*O*-(3-methyl-1-oxobutyl)-*β*-D-glucopyranosyl]oxy]-(2β,4α,15α)	CA	GB	[45]
133	19-Norkaur-16-en-18-oic acid-15-hydroxy-2-{[2-*O*-(3-methyl-1-oxobutyl)-*β*-D-glucopyranosyl]oxy}-(2*β*,4*β*,15α)	CA	GB	[45]
134	19-Norkaur-16-en-18-oic cid-2-{[3-*O*-β-D-glucopyranosyl-2-*O*-(3-methyl-1-oxobutyl)-*β*-Dglucopyranosyl]oxy}-15-hydroxy-(2*β*,4*α*,15*α*)	CA	GB	[45]
135	2β,16α,17-Trihydroxy-ent-kauran-19-oic Acid	CA	GB	[45]
136	Paniculoside IV	CA	GB	[45]
137	Mascaroside III	CA	GB	[46]
138	Mascaroside Ⅳ	CA	GB	[46]
139	Mascaroside V	CA	GB	[46]
140	20-Nor-Cofaryloside I	CA	GB	[46]
141	20-Nor-Cofaryloside II	CA	GB	[46]
142	Villanovane	CA	GB	[46]
143	Tricalysione A	CA	GB	[46]
144	2*β*,16α,17-Trihydroxy-ent-kauran-19α-oic Acid	CA	GB	[46]
145	2-*O*-(2-*O*-Isovaleryl-*β*-D-gluco-pyranosyl)-4α-atrac-tyligenin	CA	GB	[46]
146	2-*O*-(2-*O*-Isovaleryl-*β*-D-gluco-pyranosyl)-4α-atrac-tyligenin	CA	GB	[46]
147	3-O-β-D-glucopyranosyl-2-O-(2-O-isovaleryl-β-D-gluco-pyranosyl)-4β-atracty ligenin	CA	GB	[46]
148	16-O-Methylcafestol	CA	GB	[41]
149	Cafestol	CA	GB	[31]
150	16-O-Methylkahweol	CA	GB	[41]
152	Kahweol	CA	GB	[31]
152	Atractyligenin	CA	GB	[52,53]
153	2-O-β-Glucopyranosyl-atractyligenin	CA	GB	[52,53]
154	3ʹ-O-β-D-Glucopyranosyl-2ʹ-O-isovaleryl-2β-(2-desoxy-atractyligenin)-β-D-glucopyranoside	CA	GB	[52,53]
155	2-O-β-Glucopyranosyl-carboxyatractyligenin	CA	GB	[52,53]
156	3ʹ-O-β-D-Glucopyranosyl-2ʹ-O-isovaleryl-2β-(2-desoxy-carboxyatractyligenin)-β-D-glucopyranoside	CA	GB	[52,53]
157	Dehydrocafestol	CA	GB	[54]
158	Dehydrokahweol	CA	GB	[54]
159	Cafestal	CA	GB	[54]
160	Carboxyatractyligenin	CA	GB	[54]
161	Cafestol Palmitate	CA	GB	[55]
162	Kahweol Palmitate	CA	GB	[55]

SB, sun bean.

## Data Availability

Not applicable.
